# Supplementary material for: Global hypo-methylation in a proportion of glioblastoma enriched for an astrocytic signature is associated with increased invasion and altered immune landscape
Source: eLife. 2022 Nov 22;11:e77335. doi: 10.7554/eLife.77335 (PMC9681209; doi:10.7554/eLife.77335)
Supplement: Figure 2—source data 1. [file elife-77335-fig2-data1.zip › Figure_2_source_data_1/Figure_2I_J/homerResults/motif40.similar.html]

motif40

## Information for motif40

C
A
G
T
G
C
A
T
A
G
T
C
G
C
T
A
G
T
C
A
G
T
C
A
C
G
T
A
G
C
T
A
A
T
C
G
T
C
G
A
G
T
C
A
G
C
T
A
G
T
C
A
C
G
T
A
G
A
C
T
  
Reverse Opposite:  

C
T
G
A
A
C
G
T
A
C
G
T
C
G
A
T
C
A
G
T
A
G
C
T
T
A
G
C
C
G
A
T
A
C
G
T
A
C
G
T
A
C
G
T
C
G
A
T
T
C
A
G
C
G
T
A
G
T
C
A
  

|  |  |
| --- | --- |
| p-value: | 1e-23 |
| log p-value: | -5.490e+01 |
| Information Content per bp: | 1.676 |
| Number of Target Sequences with motif | 42.0 |
| Percentage of Target Sequences with motif | 5.79% |
| Number of Background Sequences with motif | 2.3 |
| Percentage of Background Sequences with motif | 0.81% |
| Average Position of motif in Targets | 99.7 +/- 48.4bp |
| Average Position of motif in Background | 131.3 +/- 19.8bp |
| Strand Bias (log2 ratio + to - strand density) | -0.2 |
| Multiplicity (# of sites on avg that occur together) | 1.11 |
| Motif File: | file (matrix) reverse opposite |

### Similar de novo motifs found

|  |  |  |  |  |  |  |  |
| --- | --- | --- | --- | --- | --- | --- | --- |
| Rank | Match Score | Redundant Motif | P-value | log P-value | % of Targets | % of Background | Motif file |
| 1 | 0.685 | A C G T G A T C A C G T A C G T A C G T G A T C G C A T A G C T A G C T A C G T G A T C G T C A C T G A C G T A C G T A | 1e-21 | -48.517458 | 5.37% | 1.02% | motif file (matrix) |
| 2 | 0.678 | C G T A G T C A C G T A A C T G A G T C G T C A C G T A C G A T T C G A G C T A | 1e-15 | -35.089788 | 5.37% | 1.16% | motif file (matrix) |
| 3 | 0.705 | C G T A C G A T C G A T C T G A T G C A C G T A C T A G T C G A C G T A C T A G T G A C G C T A C T G A C T G A C G T A | 1e-13 | -30.166420 | 4.96% | 1.40% | motif file (matrix) |
| 4 | 0.697 | C G A T C A G T G C A T A C G T A G C T G A C T G C A T G A C T G A C T G A C T G A C T G A C T G C A T G C A T G C T A C G T A C T G A C G T A G C T A C G T A | 1e-7 | -18.071016 | 2.20% | 0.61% | motif file (matrix) |
